# Making home visits: Creativity and the embodied practices of home visiting in social work and child protection

**DOI:** 10.1177/1473325016656751

**Published:** 2016-08-01

**Authors:** Harry Ferguson

**Affiliations:** School of Sociology & Social Policy, University of Nottingham, UK

**Keywords:** Home visit, ethnography, social work practice, embodiment, child protection, the senses, movement, atmospheres

## Abstract

Although the home is the most common place where social work goes on, research has largely ignored the home visit. Drawing on a participant observation study of child protection work, this article reveals the complex hidden practices of social work on home visits. It is argued that home visits do not simply involve an extension of the social work organisation, policies and procedures into the domestic domain but the home constitutes a distinct sphere of practice and experience in its own right. Home visiting is shown to be a deeply embodied practice in which all the senses and emotions come into play and movement is central. Through the use of creativity, craft and improvisation practitioners ‘make’ home visits by skilfully enacting a series of transitions from the office to the doorstep, and into the house, where complex interactions with service users and their domestic space and other objects occur. Looking around houses and working with children alone in their bedrooms were common. Drawing upon sensory and mobile methods and a material culture studies approach, the article shows how effective practice was sometimes blocked and also how the home was skilfully negotiated, moved around and creatively used by social workers to ensure parents were engaged with and children seen, held and kept safe.

Social work, as Pithouse (1998) has incisively put it, is ‘an invisible trade’ in how most of it goes on behind closed doors, primarily within the privacy of the service user's home. But while the home visit is a key practice site where social workers and service users meet, it has been largely ignored in research and the social work literature (Nicholas, 2012). There is nothing new about this neglect. Fifty years ago, Noel Timms (1964: 194) observed that ‘For a considerable period in the history of casework the home visit was largely taken for granted and no commentator seems to have considered it advantageous to describe or analyse the obvious’. Neglect of the home visit is not peculiar to the academic discipline of social work. As Pink et al. (2015: 450) point out,

home visits … are part of the everyday working life of staff across professions including nursing as well as in logistics and skilled trades, filling needs related to ageing, healthcare, deliveries, and installation or domestic work. However, … they are little analyzed in academic literatures.

This article seeks to correct for this neglect with respect to social work by drawing on the findings from ethnographic research, in which I observed social workers on home visits and audio-recorded their encounters with children and parents in cases where there were child protection concerns. Social work in families’ homes was found to be a complex activity and in attempting to meet their aims social workers and family members adopted a range of strategies. This was exemplified by a social worker who after being observed completing a sophisticated visit in which she moved from room to room, interviewing the children and parents alone and together in different combinations, described what she was doing as ‘working the house’. Because what social workers actually do on home visits and when face to face with service users, how they ‘work the house’, has barely been researched (Broadhurst and Mason, 2014), the aim of this article is to contribute to the urgent task of revealing the tacit knowledge and hidden experiences and practices of social work on home visits, so that they can be understood, conceptualised and developed.

Hall and colleagues (Hall et al., 2014; Slembrouck and Hall, 2011) have studied the nature of face to face communication between parents and practitioners on home visits by using audio-recordings to analyse talk, but without being there to observe the practice (see also Forrester et al., 2008). The research on which this article draws adopted an ethnographic approach that involved following social workers around everywhere they went and the researcher getting as physically close to practice as possible and paying attention not only to how social workers, parents and children interacted with one another through talk but also what they did, how they acted and moved (Ferguson, 2011, 2016a). A key focus was the nature of practice as a sensory and mobile experience, how it felt to journey from the office to the family’s doorstep, to enter and be in the home and the atmospheres within which encounters with families went on (Pink, 2009). Because the literature largely ignores the importance of space and the places where practice goes on (Jeyasingham, 2014) and great emphasis is placed on the power of organisational culture, managers and extensive bureaucratic tasks to shape what social workers do (Broadhurst et al., 2010), there seems to be an assumption that home visits essentially involve an extension of the social work organisation, policies and procedures into the domestic domain. The article shows the opposite to be the case, in how it was found that the home constitutes a sphere of practice and experience in its own right. As Sarah Pink's work shows (Pink, 2004; Pink et al., 2015; Pink and Leder-Mackley, 2016), the home and the practices that go on in it warrant detailed empirical and theoretical analysis. In what follows, theoretical work in social anthropology, sociology and geography will be drawn upon to argue that social workers ‘make’ home visits through the use of creativity, craft and improvisation (Ingold, 2011). Home visiting is shown to be a deeply embodied practice in which all the senses and emotions come into play and movement is central (Ferguson, 2004, 2008). Home visits require mobile embodied performances (Jenson, 2013; Urry, 2007). The article makes these arguments by, first, outlining the research study and then following the flow of the home visit as it is done, moving with practitioners from the office to the home, across the doorstep and into families' most intimate spaces, making sense of workers’ experiences and emotions as they struggle to adjust to the home, the atmospheres, feel of it, smells, sounds within it and craft meaningful and effective social work and child protection practices.

## The research study

The research was based in two local authorities in England and involved social work teams who did short-term duty and assessment work and longer term work with children and families. I did the fieldwork myself and spent three months in each local authority, being on site for two to three days per week. I went along with the social workers on their journeys to see service users, interviewing the workers about their plans and feelings. Where parents gave consent, I followed the worker into the family home and observed and audio recorded their practice encounters with children, parents and others present. On the way back to the office or on to the next visit, I interviewed the workers about their experience of the visit. Observations were also done in the social work office, and social workers and managers interviewed about their practices in general. The research was granted ethical approval by my university ethics committee and the two local authorities involved and only social workers and service users who consented were included. In total, 24 social workers were observed and audio-recorded and the research involved a total of 87 practice encounters, of which 71 were home visits, 9 were interviews with children in schools and 7 office interviews. The social workers were shadowed everywhere they went in the home, including upstairs to inspect bedrooms and interview children on their own in their bedrooms. The recruitment methods, informed consent and other sensitive ethical issues that arose are analysed at length elsewhere (Ferguson, 2016a).

## Home rules: Improvisation and the ‘making’ of home visits

To make sense of home visiting practices, it is necessary to draw on theory that analyses what the home is and the extent to which what social workers do and experience on home visits is down to their own creativity or governed by social work organisations. Many aspects of what social workers do are influenced by procedures and standardized assessment frameworks (Broadhurst et al., 2010). Particular things are expected of first visits and social workers in the research were systematic in gathering information that was informed by national and local child protection guidance, the assessment framework they worked to, asking questions about children’s health, development, parental relationships, sources of family support, other involved professionals and so on. Government guidance in England (Department for Education, 2013) requires social workers to see children who are suspected to be at risk of harm on their own as part of a ‘statutory visit’ and the research found that the family home was the most common place where they were seen.

Not only does this collection of procedures, guidance and knowledge influence the kinds of areas that are covered on home visits, it was found that the burden of administration required to keep detailed case records and report writing could have the effect of limiting the frequency with which families were visited and at times the time available to spend relating to service users on home visits (Ferguson, 2014). However, I still want to argue that what social workers do beyond the office and how they do it is not in any simple sense shaped by the technical-rational imperatives of guidance, audit and managerial surveillance. How social workers in my study used the time that they did have to spend with children and families was to a significant extent determined by them and the family and the impact of the home itself. Thus, while government policy prescribes that children at risk need to be seen on their own, there is no guidance or social work literature on where that should be done and the best timing of it during a home visit, or what is best practice in looking around a house. As Pink et al. (2015: 450) put it, people’s homes become part of workplaces, while also taking practitioners into forms of experience and knowledge that extend well beyond the reach of organisations.

The key implication of this is that there is no blueprint for home visiting. Social workers have to make their own practice by improvising their ways into and through the home. This requires practitioners to act much more on the basis of knowledge, skill, intuition, ritual and courage than bureaucratic rules and to be craftspeople and improvisers. Social workers have to ‘make’ their practice. Ingold (2011) uses the term ‘making’ to draw attention to the skill and improvisation that are easily taken for granted in practices that require craft. For instance, when a carpenter makes something by sawing wood, the strokes of the saw may look the same, but on closer examination, no two pieces of wood are identical to work with and the skill of the craftsperson lies in how they constantly have to improvise (Ingold, 2011: 216–217). The same is true for social work as no two home visits are ever the same. In their study of how people create homes that are aesthetically pleasing, comfortable and energy efficient, Pink and Leder-Mackley (2016), draw on Ingold’s concepts of ‘making’ and improvisation to show how cleverly people use lighting and furnishings to actively make the kinds of homes and the atmospheres in them they desire. To understand how home visits are made in the sense of skilfully created, our focus here must not merely be on what social workers do *in* people’s homes but on how they improvise by moving *through* the home (cf. Pink and Leder-Mackley, 2016: 172). This enables social work practice to be explored in terms of how it is made through movement as well as stillness and ‘from the encounters between people, materials and other elements of the environments of which they are part (e.g. air, light, warmth, scents)’ (Pink and Leder-Mackley, 2016: 178).

Such ‘making’ of home visits is evident, for example, in the research finding that rarely do social workers walk into homes where conditions are ideal to enable them to do the kinds of work they regard as necessary. In only a small proportion of the 71 home visits I shadowed did the workers find families all set up and waiting for them in an orderly fashion to do what needed to be done. The daily experience of family life is fluid and lively as adults and children interact, play, eat, cry, fight, the TV is on, their mobile phones ring or ping, the dog(s) bark, growl, demand attention or other distractions such as adults whose identities are often unknown to social workers are present. This flow and flux of family life provides crucial observational information that helps social workers to reach an assessment of service user welfare (Trevithick, 2012). But it also threatens to be disruptive of the conditions required to conduct meaningful social work, and sometimes workers step into actual chaos, which is in stark contrast to the kind of control the practitioner has and the kind of order that is possible in the social work office or consulting room.

On home visits, the conditions that enable meaningful work to be done have to be *made* and the following field notes from a visit provide a typical example of the craft involved in how social workers met the challenge:

Mother (‘Lisa’) answered the door. As soon as the social worker stepped into the home, the dog was jumping up to try and escape outside and Lisa was roaring at and hitting it to try and stop it. The social worker walked into the dining area and was met by 5-year-old Leon doing dangerous swings from the bannisters of the stairs and his 7-year-old sister soon joined in. The dog came into the room and was tearing round and chewed up an entire cushion. The social worker had negotiated with Lisa prior to the visit to spend some time alone with the children but there was nowhere she could do so. The small bedrooms were too cluttered and there were no armchairs or settee, so at the social worker’s request the dining room table and chairs were set up.

Lisa left and the worker then did 20 minutes work with the children. She worked hard to hold their attention and used a drawing exercise through which both children spoke about family relationships and their feelings.

Tellingly, the social worker commented afterwards on how much calmer it was today and how delighted she was to have got what she estimated was 15 minutes of good work done with the children. Through improvisation, movement, adept use of the space and the cooperation of the service users, the worker managed to create enough order to enable some meaningful work to be done with the children and parent.

If the home visit is to be fully understood, all aspects of movement, transitions (office to neighbourhood; doorstep into the home), ritual and the body and senses in action must be described, analysed and theorised. Thus, while at its most obvious, a house is a physical structure, a central insight of theoretical work on the home is that it should not be regarded as a neutral space, which is little different to being on the street, in the office or other public buildings. When social workers home visit, they step across a boundary into another world, a process that can be understood in terms of what anthropologists call ‘liminality’. This is an ‘in-between’ state, a sense of normlessness that arises from moving from one state to another (Turner, 1969). The doorstep, hallway and porch carry danger, because they straddle the threshold between public and private worlds, they are neither in nor out. Within them, we experience the world differently, we quite literally do not know where we stand. This is why hallways so often have mirrors, so that we can glance at ourselves to reconnect with who we are (Rosselin, 1999). This was borne out in the research by how entering the home could be a liminal experience for workers, causing a sense of disorientation that lasted for the opening minutes and that in some cases persisted for the duration of the entire visit.

The anthropologist Daniel Miller argues that we need to think about the house as having ‘agency’, by which he means it is an object that influences how we act, that acts upon us as well as us acting upon it. ‘Things do things to us, and not just the things we want them to do’ (Miller, 2010: 94). The home is a central part of the objects, the ‘material culture’, that make up our lives and what we are. Material culture studies argue that unless critical attention is paid to such objects we will fail to account for the power they have over what people do. Scholar (2016) argues convincingly that it is crucial that far more attention is paid to the artefacts and objects that make up social work. Miller (2010) also argues that the closer people get to everyday objects the closer their relationships are with people. Thus, the research sought to explore how the home constrains social workers’ practice and also how getting to know the home, having a relationship with it as it were, helps to make best practice. The use of Pink’s (2009) method of ‘sensory ethnography’ enabled the research to get close to practitioners’ embodied experiences of smells, noise, touch and evoke the influence of how a place and experience *feels*. A further dimension of this is the growing interest in the social sciences in *affect atmospheres*. As Teresa Brennan has put it, ‘Is there anyone who has not, at least once, walked into a room and “felt the atmosphere”?’, and she goes on to explore how ‘one person can feel another’s feelings’ and the energy that is in a room (Brennan, 2004: 1).

While atmospheres, the *feeling* of a place and the internal world of families and the home have been largely ignored in the social work literature, in the early 1970s, Helen Harris Perlman tried to capture them through the notion of ‘inner space’:

As for the content of our study, it needs to be a more precise and detailed accounting of man’s [sic] ‘inner space’. The inner space I refer to is of two sorts: one is within the individual man, contained inside his skin, yet revealing itself always by his words and actions, and penetrated always by the words and actions of other people and circumstances; the second is the inner space of the family life – the emotional climate that is contained within the walls of a room or a household, constantly in play among, upon, and within its members. (Perlman, 1971: 192)

This establishes an important agenda for inquiry into the internal lives of social workers, as they make home visits and navigate the atmospheres and inner space of domestic/family life. To meet their aims, workers must be craftspeople, using skilful improvisation in ‘working the house’.

## Getting to the home: Into the unknown

The work involved in home visiting begins well before the practitioner enters the actual home, as prior to setting off from the office they imagine and anticipate what might happen. Risk assessing and planning the visit goes on with managers and after that by the worker (usually alone) during the journey to the home. Social workers can never be sure what they are going to walk into and being able to tolerate the unknown and respond spontaneously to the unexpected is a key part of the job. Social workers do two kinds of visits: announced, where they are expected, and unannounced, where they visit without prior warning. Going into the unknown is the source of some excitement but also anxiety and fear. This is exemplified by a social worker in the car on the way to an announced first visit:

you’re going into an unknown situation and you don’t know what’s going to happen and you don’t know how people are going to react to you. I mean I’ve never met these parents before, I can see on [the case record system] that Dad has got previous sort of behaviour issues, so it makes me think, oh, is he going to sort of take that out on me?

Jeyasingham (2014) cautions against over dramatizing and sensationalising everyday practice that he suggests is in reality mostly ‘mundane and quotidian’, that is unremarkable. However, this invalidates key aspects of the underlying existential experience of social work. Day to day home visiting may indeed become *routine* and often be unremarkable in the sense of lacking high drama, but it is still routinely alive with the possibilities of the unknown, full of complex challenges and emotions and sometimes drama.

The unexpected occurs even on announced visits to families who social workers know well, which is exemplified by a case involving ‘Rita’, 18, and her 13 months old son, who was on a Child Protection Plan due to domestic violence. Also resident in the home were Rita’s mother, sister and two brothers. The social worker regarded it as a routine visit to see how Rita was coping and to help her sustain a child care routine, but as recorded in my field notes the plan quickly had to be abandoned.

Rita was waiting for the social worker at the front door and was shouting at him in an angry upset way as he approached the front gate and walked down the path. She’d had a row with her sister and mother and was shouting about wanting to get them out of the house. The row continued for the first 10 minutes of the visit and the social worker put all his energy into successfully calming things down. Rita kept appealing to the social worker to get her out of the house and into a refuge.

The social worker had to completely revise their plans and improvise on the spot as the entire visit consisted of him skilfully helping Rita and the other family members to resolve their arguments and discussing options.

## Entering the home: Ritual and the full engagement of the body and senses

Gaining access is sometimes far from straightforward and on a small number of visits in the study entry to the home was initially denied, but the practitioners subsequently got in. An exception was where a social worker was kept on the doorstep for 14 minutes and never allowed entry to the home by a mother who had already had five children removed into care. She was angry, fearful, sad and tearful, demanding reassurance that her one child who remained living with her would not be taken into care.

Contrary to the implication in much of the literature that practice in the home is an extension of the organisation, what goes on in the home visit is characterised by its difference from the experience of working in the office. The journey from the office onto the streets and into the home requires workers to make a transition from a static experience dominated by being seated in front of the computer screen and based on use of the mind rather than the body, to an experience that is mobile, deeply sensory and embodied. A case example that typifies what the data show about the embodied and sensory nature of home visiting and the struggles involved in becoming oriented to the home and a family’s inner-space involved a home visit to a 9-year-old boy I will call John regarding concerns about him allegedly being hit, neglected and emotionally abused. The social worker had visited for the first time a week earlier and found what she regarded as very poor home conditions, spoke to John on his own in his bedroom and told the parents that the home needed to be cleaned up. On arrival, outside the home, there were six young men on the street drinking bottles of lager, one sat on the family’s gate, who took a few minutes to move and only did so after asking the social worker ‘who are you?’. The garden was strewn with litter and discarded objects like old bicycle wheels. Mother – who I call as Mrs Jones – answered the door and did not make eye-contact. On stepping into the home, a pungent odour was immediately obvious and in the sitting room the curtains were drawn, despite it being a hot sunny day, adding to a musty, dark atmosphere. John was sat on one armchair and his mother on the armchair opposite and the social worker snuggled up beside John. Martin, a 17-year-old cousin of John’s, was sat on the settee and was naked from the waist up. He never acknowledged our presence or looked up from his mobile phone. The atmosphere was awkward, tense. The social worker struggled to find her composure, as she sat on the edge of the armchair, she asked questions awkwardly of Mrs Jones and John, while ignoring Martin. After four minutes of this, the social worker suddenly suggested that she look around the home and Mrs Jones led the way. As we walked out of the sitting room through the doorway, which had no actual door, two huge dogs were jumping up from behind a half door in the kitchen. Father arrived home simultaneously and he and Mrs Jones commented affectionately on the dogs, saying how harmless they were. This was not convincing.

The social worker climbed the stairs and went first to Martin’s room. It had no door but a cloth covering, which Mrs Jones held back to allow access. It was quite dark as a sheet covered the window, sparsely furnished, with a bed with covers. She then went into John’s room, which again had no door. There were piles of clothes, bedclothes and other things stacked high covering almost up to the top of the window and hanging over the boy’s bed. The social worker asked various questions of Mrs Jones, in a sensitive manner, trying to get her to explain what they’d spent the past week doing and what all of this stuff was. Returning to the landing, the third bedroom was blocked off. There did not appear to be a door but something more solid and permanent. The social worker asked if she could see into it and Mrs Jones shrugged it off making it clear that she was not going to see any more.

While going downstairs, the worker asked to see the kitchen. She did not get to see it last time due to fear of the dogs, who were put outside today to permit access. Martin was in there now having a smoke. The social worker had a good close up look at the cooker and work surfaces, and asked about where the dogs go to the toilet. She then made her way back to the sitting room. There was movement in it now, John was on a scooter. Father was sat where Martin had been on the settee and was pleasant. The social worker returned to her original seat. Something had lifted. The action of looking around had shifted something, moved things on; rearranged things. The social worker seemed less burdened, more coherent and in control. She had gained her composure, confidence and authority and a 40-minute child-centred discussion ensued.

Afterwards, the social worker said that while it was always her intention to look around the home, she did not intend to do it so soon. On our terms, she had to improvise. She said she often finds the first five minutes of such encounters difficult. Within the terms being used here, her immediate liminal experience of the home caused disorientation, which was heightened by the emotional and sensory impact of the home and the people present. She felt anxious about Martin, who she knew had been aggressive to other social workers in the past. So the initial menacing atmosphere compounded her anxiety. But she stressed that it wasn’t just fear for her own safety that accounted for her feeling of discomfort, but what she described as the foul smell, sticky carpet, lack of light and fresh air, which made being in the sitting room very difficult; an experience the social worker afterwards kept referring to as ‘depressing’. The social worker found the whole atmosphere in the home almost overwhelming and unbearable, describing it as like ‘stepping into another world’.

By the social worker’s account, and interpreted through the lens of material culture studies (Miller, 2010; Scholar, 2016), this was a home that threatened to over-power, its agency was large in how it presented as an object in its own right and the resulting atmosphere and feelings of anxiety and disgust threatened to immobilize her. However, this social worker managed to overcome these feelings and her disorientation and inertia by *moving*. Her normal pattern was to leave looking around the home to later in the visit, but in this case, she felt compelled to move and recognised how it helped, in every sense, to move things on.

because it can be quite intense when you’re sat there opposite somebody, and you might have a walk around and then say: ‘Oh this is so and so’s bedroom and blah, blah, blah’, and then it might be a bit easier to then come down and continue with the rest of the interview. … Sometimes I think, yeah, just physically getting around and getting up and moving around a bit can feel a little bit easier, maybe a little bit less intense, people shift around a bit in the room when you come back, you know.

This evidence fits with Jeyasingham’s (2014) argument that space is not just a backdrop to social worker’s movements and activities, but participants are involved in *producing* space and creating opportunities for thinking about things in different ways. Thus, in this case, for example, a new experience of space in this home became possible. The social worker’s capacity to improvise through movement was a key part of her craft and attempt to wrestle control of the situation from the home and was a clever way to buy some time to think about how to address the presenting situation, such as what to do about Martin’s presence. She felt ‘relieved’ when he wasn’t in the sitting room when she returned to it. The worker had stepped into a forbidding affective atmosphere in which she could *feel* family members’ resentment and possibly shame about her presence and once she moved through the home her actions changed the atmosphere, making it more accommodating. She was able to get control of the home, to see most of it and conduct the interview she wished to.

Instances were observed in the research where social workers did not manage to come to terms with the home and their visceral experience of it. After a 45-minute visit, a social worker admitted that she had failed to effectively engage with the children and challenge a father about neglect concerns and the first thing she said on emerging from the home was:

As soon as I walked in the house, I just felt utterly uncomfortable. I don’t know. There were lots of things I didn’t say or do that I think, I think the dirty house just kind of overtook me, to be honest. I’ll have to go back because I don’t believe half of it … I just felt uncomfortable with the house as soon as I went in.

Feeling unable to settle into the home by bringing their emotional and visceral experiences – in this instance, of disgust – under control caused some workers to be overwhelmed by it and their purpose and ability to engage with children and parents in skilful, authoritative ways flew out the window (see Ferguson, 2016b).

Such visceral experiences and strong emotions were not confined to ‘neglectful’, so called ‘dirty’ homes and poverty stricken families. All home visiting is embodied, irrespective of what kind of a home it is. Clean, well-presented homes presented their own challenges, especially when occupied by middle class families. After a visit to a spotless middle class home where the infant was on a child protection plan, due to suspected physical abuse, the social worker complained in the research interview about often running out of things to talk about. The spotlessness somehow emptied the encounters of meaning, leaving little material for the worker to get hold of.

## Bedrooms and kitchens: Working in the heart of inner-space

It was commonplace for social workers to go right into the heart of families’ inner-space into their bedrooms, bathrooms and kitchens. On 40 of the 71 home visits, I observed social workers went upstairs and inspected the bedrooms. In most of the 31 visits where this did not happen when I was present, the workers had been upstairs on a visit prior to the research visit or did so on the next one. On 9 of these 40 bedroom visits they did not merely do inspections, but children were seen on their own and worked with alone for a period in their bedroom. On another six visits children were seen on their own in other parts of the home- the sitting-room, kitchen or garden. Inspections illustrate how the culture of practice in social work and child protection was found to be very authoritative. However, the use and meaning of various rooms in practice was also found to be based on attempts to establish empathy with children and parents through their possessions, such as toys, photos and other stuff. Bedrooms are a key place where such personal objects are found and that was one reason why some social workers choose to work on their own with children in them. In this manner, the creative ways in which social workers worked the house involved them in using it and other objects it contains to get closer to people to try to find enough out about their lives to promote their well-being and safety.

This is exemplified by a case that involved three children, here called Justin (6), Florence (5) and Liam (3). The referral that led to this phase of social work involvement concerned bruising on Justin, allegedly by his step-father Neil who had been living with Emma, the children’s mother. Neil had moved out and a new boyfriend, Gareth, had moved in. Gareth had a history of domestic violence offences for which he had recently served a prison sentence. The social worker had been involved with the family for a couple of months and was meeting Gareth for the first time on this visit, a key aim of which was to assess what risk he represented to Emma and the children.

On arrival, the social worker, who I shall call Lynne, went into the sitting room where she excitedly spoke to Florence and sat on the settee beside Justin. Lynne held Florence and nestled her into her while she related to Justin who gathered himself up in the corner of the settee. Emma was standing close by and Gareth then entered the living room and Lynne got up, walked towards him and introduced herself, including initiating a handshake. The social worker said she wanted to see Emma in the kitchen alone and negotiated with the children that she’d spend time with them later. She gave them some pens and paper she had brought along to play with. The visit lasted 46 minutes, four of which were at the start with the children in the sitting-room and five in the kitchen with Emma alone, catching up on issues to do with Neil. Lynne then requested to see Gareth on his own and decided that the garden was the best place to do that and spent six minutes there alone with him. She revealed afterwards that she chose to do this early on in the visit to check with him what Emma knew about his violent past (he said she knew everything), to ask him if he was still using drugs (he said he wasn’t) and also so that she could check out information that he keeps a dangerous dog hidden there. She cleverly chose the garden not just to create confidential space away from Emma and the children but so that she could see whether he was keeping the dog there (he wasn’t). Lynne then asked to see Emma and Gareth together in the kitchen where she spent six minutes authoritatively discussing her concerns about the risk of domestic abuse. Lynne followed this by spending 13 minutes in the bedroom with the children, alone, exploring among other things their feelings towards Gareth. This was followed by another six minutes with the adults together in the kitchen reiterating her concerns about the risks of violence and the harm and consequences that can occur should it happen. She then spent four minutes with Emma alone in the kitchen, which included checking out her vulnerability and whether she was experiencing any domestic abuse and, finally, two minutes saying goodbye to the children.

This example involved some of the most mobile practice in the study, but it typifies the commonplace ways that practitioners conducted what were regarded as effective home visits: by moving through the home, the clever use of space to see individuals alone and in various combinations in different rooms and the garden and the improvisation and agility this required. Even the 13 minutes this worker spent with the children in the bedroom involved a mixture of being seated and still and some moving around as the children got up onto the window sill and the window was open and a danger to them. The worker was twice sat on the bed, twice at the window sill, and once sat on the floor, all the while communicating with the children and using objects like toys, pens and paper, as well as talk in the process. The craft involved in such skilful communication was also enacted through embodied practices of touch – like when the worker nestled the children close to her when sat on the settee and initiated the hand shake with Gareth.

Afterwards, I asked the worker about what I spontaneously referred to as her ‘choreographed’ approach and what lay behind how she moved around and saw different family members in different combinations and rooms:

SW: Yeah, but I didn’t have that planned in my head, though, because you don’t know what you are walking into, so you have to be able to, it is just all very spontaneous.

HF: But there must be a kind of a knowledge base behind it?

SW: Yeah, well experience, knowing how to work a house, a family, kids and to check, you know check … It’s how to work a house, how to work a family.

What the worker describes as ‘just all very spontaneous’ is another way of articulating what this article is calling improvisation. And as she also notes, it is the conditions of uncertainty that home visiting routinely involves that demand it (see also Pink et al., 2015). ‘Working the house’ is a way of trying to give expression to social workers’ and family members craft in using the home to achieve their aims. Another manifestation of this was how some adults evidently sought to use the home to block social worker’s movements and access to things they wished to keep private or conceal. The Jones case above where the mother blocked the social worker from seeing the third bedroom is a case in point. Another pattern in such resistance was exemplified on a visit where the social worker asked to see where the baby sleeps and was taken to the parents' bedroom which had a cot in it, positioned beside the double-bed. The father entered the bedroom first and positioned himself in a way that blocked the social worker from moving beyond the doorway and she could only gain sight of the child’s cot from a distance. The father never said anything that expressed resistance, he used the house to help him embody it.

The findings also reveal the limitations to the home as a site for safe practice. The presence in the house of parents or other people who have harmed children or could be a threat to them mean that power dynamics in homes can leave children too fearful and inhibited to speak truthfully about their experiences. Social workers too, shut away in the bedroom with the children, can feel captives to fearful and resentful parents and never manage to fully relax into the direct work with the child and gather their story. Social workers told many stories of parents being suspected of listening outside of doors. Some encounters in the research were terminated and cut short by parents, who initially gave their permission for the worker to see their child alone and then walked into the room or made noise outside it to signal that it needed to end. This is why many social workers and managers in the research understood the need for children also to be seen in other places, such as schools, offices, clinics, family centres, cafes and in the car. But for reasons of speed, efficiency and in some instances simply not being aware of its limitations, some children were seen only at home.

Bachelard (1969) uses the term ‘reverberation’ to describe the sensuous nature of domestic space and how the characteristics of houses and those in them physically affect us and become part of us. The data support the argument I have made in detail elsewhere (Ferguson, 2011) that the deeper practitioners get into the intimate spaces of the home, like bedrooms, the greater the risk of being affected by the reverberations of the lives of those who live there. This means that the risks of experiencing the disorientation of liminality are not confined to entering the home but can occur when the worker is involved in moving from one room to another and crossing thresholds within the house. Workers were observed, for instance, hesitating on landings before asking to enter a closed bedroom door, or getting the encounter in (and with) the bedroom over as quickly as possible. These dynamics were also evident in social workers *avoidance* of bedrooms and other intimate spaces. Some in the study (a minority) refused to go upstairs, because they did not see it as a legitimate site for practice, either because for them it is too intrusive or because they do not feel safe alone with children there, fearing they could be the subjects of allegations of sexual abuse. These are legitimate worries, but the basis for avoidance of intimate spaces is also deeply personal and caused by the uncomfortable feelings that reverberate within individual social workers when they step into the deepest recesses of the family’s life space and which can make carrying out such actions unbearable. The cost of such avoidance is the distance it puts between the worker and reaching as deep as possible an understanding of the children and other family member’s lived experiences and safety, which is what they are there to do.

## Conclusion

Despite the existence of a large social work research literature, the knowledge base concerning home visiting is at an early stage of development. What is needed is nothing less than a social science of the home visit. This article has tried to contribute to this by drawing on methods of mobile and sensory ethnography, material culture studies and concepts of liminality, making, craft and improvisation to help reveal the hidden experiences, tacit knowledge and practices that go on when social workers conduct home visits in child protection work. This is intended to provide greater clarity about what social workers do and a language and theoretical resources to help make sense of the core processes, skills and embodied practices of home visiting, so that good practice can be recognised and developed.

I have argued that while social work practice is always influenced by organisational imperatives the home visit constitutes a domain of experience and practice in its own right. Every visit and contact with service users requires the worker to come to terms with the particularities of this individual home, family, situation and to reflect deeply on their experience of social work as a mobile embodied practice. To overcome the disorientation that can occur on stepping into the home and be able to manage the powerful reverberations within the self and emotions like anxiety and disgust, practitioners need to critically reflect on what they are experiencing and perform the balance of stillness and movements that are essential to effective home visiting. Crucially, on returning to the office workers need to receive supervision that is attuned to their emotional and visceral experiences and the impact of the home itself. The research findings bear out Miller’s (2008, 2010) argument that the closer people get to everyday objects the closer their relationships are with people. As Scholar concludes, in social work objects ‘have an inherent capacity to “act”, sometimes beyond or irrespective of the symbolic meanings that we ascribe to them. They do so in influencing the manner in which practice is undertaken, and by making certain things possible and others difficult or impossible’ (Scholar, 2016: 13). And there is no more significant non-human object in people’s lives than their home. A deep understanding of the nature of home visiting as an embodied experience is required, how the home can be used as a resource for creative change, and how this occurs through the purposeful use of movement, craft, skill and improvisation that are at the heart of face to face practice and making home visits.
